# Combination of Static Echocardiographic Indices for the Prediction of Fluid Responsiveness in Patients Undergoing Coronary Surgery: A Pilot Study

**DOI:** 10.3390/jcm10091886

**Published:** 2021-04-27

**Authors:** Hye-Bin Kim, Sarah Soh, Jong-Wook Song, Min-Yu Kim, Young-Lan Kwak, Jae-Kwang Shim

**Affiliations:** Department of Anesthesiology and Pain Medicine, Anesthesia and Pain Research Institute, Yonsei University College of Medicine, Seoul 03722, Korea; kakddugi@yuhs.ac (H.-B.K.); yeonchoo@yuhs.ac (S.S.); sjw72331@yuhs.ac (J.-W.S.); minyu92@yuhs.ac (M.-Y.K.); ylkwak@yuhs.ac (Y.-L.K.)

**Keywords:** left ventricular end-diastolic area, stroke volume index, Frank-Starling mechanism, cardiac preload, fluid responsiveness, echocardiography, doppler

## Abstract

We investigated the role of echocardiographic indices consisting of left ventricular end-diastolic area (LVEDA) in combination with Doppler-derived surrogates of diastolic compliance and filling (E/E′, E′/S′, E′/A′; early transmitral flow velocity (E), tissue Doppler-derived early (E′) diastolic, late (A′) diastolic, or peak systolic (S′) velocity of the mitral annulus) in predicting fluid responsiveness in off-pump coronary surgery. Hemodynamic and echocardiographic variables were prospectively assessed under general anesthesia before and after a fluid challenge of 6 mL/kg during apnea at atmospheric pressure in 64 patients with LV ejection fraction ≥40%. Forty patients (63%) were fluid responders (≥15% increase in stroke volume index). E/E′ and E′/S′ could predict fluid responsiveness with area under the receiver operating characteristic curve (AUROC) of 0.71 (95% confidence interval [CI], 0.56–0.85; *p* = 0.006) and 0.68 (95% CI, 0.54–0.82; *p* = 0.017), respectively. The combination of LVEDA and E/E′ showed incremental predictive ability for fluid responsiveness compared with LVEDA (AUROC, 0.60; *p* = 0.170) or pulse pressure variation (AUROC, 0.70; *p* = 0.002), yielding the highest AUROC of 0.78 (95% CI, 0.66–0.90; *p* < 0.001). The combined index of echocardiographic variables reflecting LV dimension (LVEDA) and diastolic compliance and filling (E/E′) is a potentially useful predictor of fluid responsiveness.

## 1. Introduction

For perioperative and critical care, appropriate fluid resuscitation guided by reliable preload indices is of pivotal importance as only half of the patients are fluid responsive [1] and superfluous fluid administration actually leads to increased mortality [2]. So far, emerging evidence advocates the ability of dynamic preload indices, such as pulse pressure variation (PPV) or stroke volume variation, to determine a patient’s status on the Frank–Starling curve and thus, fluid responsiveness [3]. However, these dynamic indices are subject to limitations related to heart–lung interaction and arrhythmia [4], which may preclude their use in patients receiving lung-protective ventilation or those with a rhythm other than sinus, spontaneous breathing efforts, pulse pressure hypertension or pulmonary hypertension [5,6].

By contrast, the assessment of static preload indices is feasible regardless of heart rhythm or heart–lung interaction, while they are unable to predict fluid responsiveness. Indeed, the use of invasively acquired measures of filling pressures using a pulmonary artery catheter (PAC) for assessing fluid responsiveness has been discouraged [7]. Similarly, the predictive ability of a static echocardiographic index, left ventricular end-diastolic area (LVEDA), for fluid responsiveness is also poor [3]. On the other hand, echocardiographic measurements of combinations of early transmitral flow velocity (E), tissue Doppler-derived early (E′) diastolic, late (A′) diastolic, or peak systolic (S′) velocity of the mitral annulus may provide surrogate information regarding LV filling pressure (E/E′), preload (E′/S′) and stiffness (E′/A′) [8,9,10,11].

Hypothetically, combining LVEDA and these indices would provide more insights regarding the LV dimension, as well as diastology (compliance and filling) that governs the ventricular response to a fluid challenge [11], which could be used to identify fluid responders. Moreover, these values can be reliably obtained non-invasively regardless of respiratory status or heart rhythm (except for A′). The primary aim of this prospective trial was to investigate the role of echocardiographic indices, including LVEDA, combined with Doppler-derived parameters of diastolic compliance and filling in predicting fluid responsiveness in patients undergoing surgical coronary revascularization.

## 2. Materials and Methods

### 2.1. Participants

The current study was conducted at Severance Cardiovascular Hospital, Yonsei University Health System, Seoul, Republic of Korea, after being approved by the institutional review board (IRB number: 4-2017-0403) and registered at the clinicaltrials.gov (NCT03222778) before recruitment started. This study was conducted following the Strengthening the Reporting of Observational Studies in Epidemiology (STROBE) guidelines and according to the Declaration of Helsinki. A total of 66 patients scheduled for off-pump coronary surgery between August 2017 and March 2020 were enrolled after obtaining written informed consent. The exclusion criteria were kidney disease requiring renal replacement therapy, heart rhythm other than sinus, LV ejection fraction <40%, lateral wall motion abnormalities, or any valvular heart disease of ≥moderate severity.

### 2.2. Anesthetic Management

All subjects received standardized anesthetic care as previously described [12]. In brief, anesthesia was maintained with the continuous infusion of sufentanil and sevoflurane and neuromuscular blockade using rocuronium. The ventilator was set to deliver 8 mL/kg (ideal body weight) of oxygen at a respiratory rate of 8–14 breaths/min, I:E ratio of 1:2 and a positive end-expiratory pressure of 5 cmH_2_O. The inspired oxygen fraction was 0.4 with air. A PAC and transesophageal echocardiography (TEE) probe were inserted in all patients and the former was connected to a Vigilance II monitor (Edwards Lifesciences LLC, Irvine, CA, USA) assessing continuous cardiac output. PPV was acquired from the radial arterial pressure waveform connected to Philips Intellivue MP70 (Philips Medical Systems, Suresnes, France). The target mean arterial pressure during surgery was 60–80 mmHg, which was maintained by administering norepinephrine, vasopressin (added when the norepinephrine requirement exceeded 0.3 μg/kg/min), or nicardipine as necessary.

### 2.3. Study Protocol

Assessment of hemodynamic and echocardiographic variables was performed 15 min after the insertion of a PAC under general anesthesia (baseline) and 10 min after the completion of fluid challenge. A fluid challenge of 6 mL/kg (ideal body weight) was performed for 10 min using 6% balanced hydroxyethyl starch 130/0.4 (Volulyte^®^; Fresenius Kabi, Bad Homburg, Germany).

Hemodynamic variables included mean arterial pressure, heart rate, PPV, central venous pressure (CVP), pulmonary artery occlusion pressure (PAOP), cardiac index and stroke volume index (SVI). PPV was obtained as an average of 4 cycles of 8 s. Filling pressures were measured during apnea at atmospheric pressure. For cardiac index and SVI, the average of 3 serial STAT mode measurements was recorded.

Echocardiographic variables included LVEDA, E, E′, A′ and S′. They were all measured with TEE during apnea at atmospheric pressure by a single examiner (J-K Shim). LVEDA measurements were obtained from the transgastric mid-papillary short axis view and E, E′, A′ and S′ measurements were obtained from the midesophageal 4-chamber view. An average of 3–5 beats was recorded for LVEDA (papillary muscle excluded in tracing the leading edge of the endocardium; automatically computed by Siemens speckle-tracking algorithm (ACUSON SC2000 PrimeTM, eSie VVITM, Siemens Medical Solutions USA Inc., Mountain View, CA, USA)), E, E′, A′ and S′. Pulsed-wave Doppler measurement of E velocity was performed between the mitral leaflet tips. Tissue Doppler imaging (TDI) measurements of the E′, A′ and S′ were carried out at the lateral mitral annulus (sample volume, 2–3 mm; sweep speed, 50–100 mm/s). LVEDA and E, E′, A′ and S′ values were all measured thrice each and the average value was used for analysis. Surgical incision was deferred until the completion of the last measurement. Measurements of the echocardiographic variables were conducted by J-K Shim who was blinded to the data on hemodynamic variables. Vasopressor requirement and plateau inspiratory pressure at the time of measurement were also recorded.

### 2.4. Study Endpoints

The primary endpoint was to assess the predictive abilities of the combined echocardiographic preload indices consisting of LVEDA and Doppler-derived parameters of diastolic compliance and filling (E/E′, E′/S′, or E′/A′) for fluid responsiveness (defined as a ≥ 15% increase in SVI). The secondary endpoint was to assess the predictive abilities of PPV, CVP, PAOP and LVEDA on fluid responsiveness and compare the predictive abilities of the combined echocardiographic preload indices with those of LVEDA or PPV alone.

### 2.5. Statistical Analysis

Statistical analyses were conducted using SAS (version 9.4; SAS Inc., Cary, NC, USA) and SPSS for Windows (Version 25; SPSS Inc., Chicago, IL, USA). Sample size was predicted under the assumption that the area under the receiver operating characteristic curve (AUROC) of the combined echocardiographic index would be greater by 0.2 than the previously reported AUROC of 0.64 of LVEDA alone [3], to aim for an AUROC of greater than 0.8, which is usually considered as having a good predictive ability. Thus, 60 patients were required to obtain 80% power (alpha 0.05) assuming a fluid responsiveness rate of 60% [12] and a total of 66 patients were enrolled accounting for an additional drop-out rate of 10%.

Intra-observer variabilities of the measured echocardiographic variables were assessed using the intra-class correlation coefficient by a one-way random effects model. An intra-class coefficient of more than 0.85 usually indicates that the measurement is consistent.

Continuous variables were first assessed for their normality using the Kolmogorov–Smirnov test. Intergroup comparisons of continuous variables between fluid responders and non-responders were performed using the independent *t*-test or Mann–Whitney U test according to the results of the normality test. Intergroup comparisons of categorical variables between fluid responders and non-responders were conducted using the chi-square test or Fisher’s exact test. Intragroup comparisons of continuous variables at baseline and after fluid challenge in both responders and non-responders were carried out using the paired *t*-test or the Wilcoxon signed-rank test according to the results of the normality test.

The AUROC was calculated for the potential candidates of preload indices to test their predictive ability for fluid responsiveness. To investigate the incremental value of combining echocardiographic indices for predicting fluid responsiveness, we performed multivariable logistic regression analysis and the AUROCs of the tested variables were compared using the DeLong test. A nomogram was constructed using a combination of echocardiographic indices showing the highest AUROC in the multivariable analysis.

Data are presented as mean ± standard deviation (SD), median (25–75% interquartile range (IQR)), or *n* (%). A value of *p* < 0.05 was considered statistically significant.

## 3. Results

The PAC could not be properly positioned in two patients who were then excluded from the data analysis; thus, data from only 64 patients were analyzed. A total of 40 patients (63%) were fluid responders. The intra-class coefficients of echocardiographic variables that were measured thrice and averaged were 0.96, 0.98, 0.96, 0.94 and 0.96 for LVEDA, E, E′, S′ and A′, respectively, indicating excellent reproducibility. Intergroup comparisons of patients’ characteristics between responders and non-responders are displayed in Table 1. None of the patients required norepinephrine infusion in excess of 0.3 μg/kg/min (median dose requirement 0.04 μg/kg/min).

Intergroup comparisons of hemodynamic variables and invasive indices of preload between responders and non-responders are displayed in Table 2. At baseline, no significant intergroup differences were found in the assessed variables including CVP and PAOP, except SVI and PPV, which were significantly lower and higher, respectively, in the responder group than in the non-responder group. After the fluid challenge, all variables showed significant changes from their corresponding baseline values, except cardiac index and SVI in the non-responder group.

Intergroup comparisons of echocardiographic indices of preload between responders and non-responders are displayed in Table 3. At baseline, no significant intergroup differences were observed in the assessed variables including LVEDA, except E/E′ and E′/S′, which were lower and higher, respectively, in the responder group than in the non-responder group. After the fluid challenge, all variables showed significant changes from their corresponding baseline values, except E′/A′ in the non-responder group.

**Figure 1:**
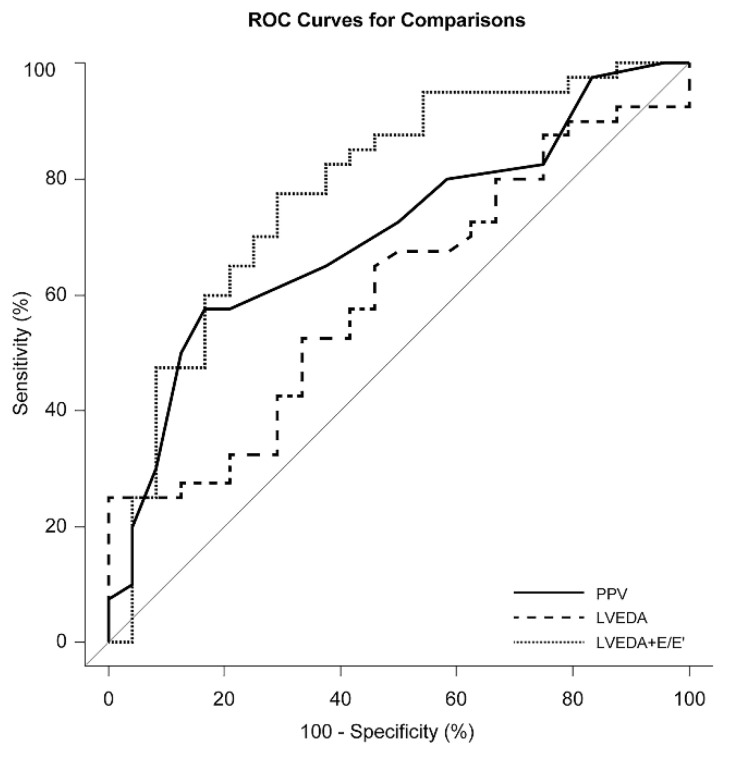
Comparison of receiver operating characteristic (ROC) curves of pulse pressure variation (PPV; solid line; AUROC, 0.70; 95% CI, 0.57–0.83; *p* = 0.002), left ventricular end-diastolic area (LVEDA; dashed line; AUROC, 0.60; 95% CI, 0.46–0.74; *p* = 0.170) and combination of LVEDA and the ratio of early transmitral flow velocity to early diastolic velocity of the mitral annulus (LVEDA + E/E’; dotted line; AUROC, 0.78; 95% CI, 0.66–0.90; *p* < 0.001). Abbreviations: AUROC, area under the ROC curve; CI, confidence interval.

The results of ROC curve analysis of the potential candidates of preload indices are displayed in Table 4. Among the invasive indices of preload, only PPV was able to predict fluid responsiveness with an AUROC of 0.70 (95% CI, 0.57–0.83; *p* = 0.002). Among the echocardiographic indices of preload, E/E′ and E′/S′ could predict fluid responsiveness with AUROCs of 0.71 (95% CI, 0.56–0.85; *p* = 0.006) and 0.68 (95% CI, 0.54–0.82; *p* = 0.017), respectively. The combination of LVEDA and E/E′ in the multivariable model showed incremental predictive ability for fluid responsiveness compared with LVEDA or PPV alone (Table 4 and Figure 1), yielding the highest AUROC of 0.78 (95% CI, 0.66–0.90; *p* < 0.001). Power calculation from the observed results yielded a power of 87%. The AUROC of the combination of LVEDA and E/E’ was significantly larger than that of the LVEDA alone (*p* = 0.020) while the AUROC of the PPV was not when compared to that of the LVEDA (*p* = 0.326).

**Figure 2:**
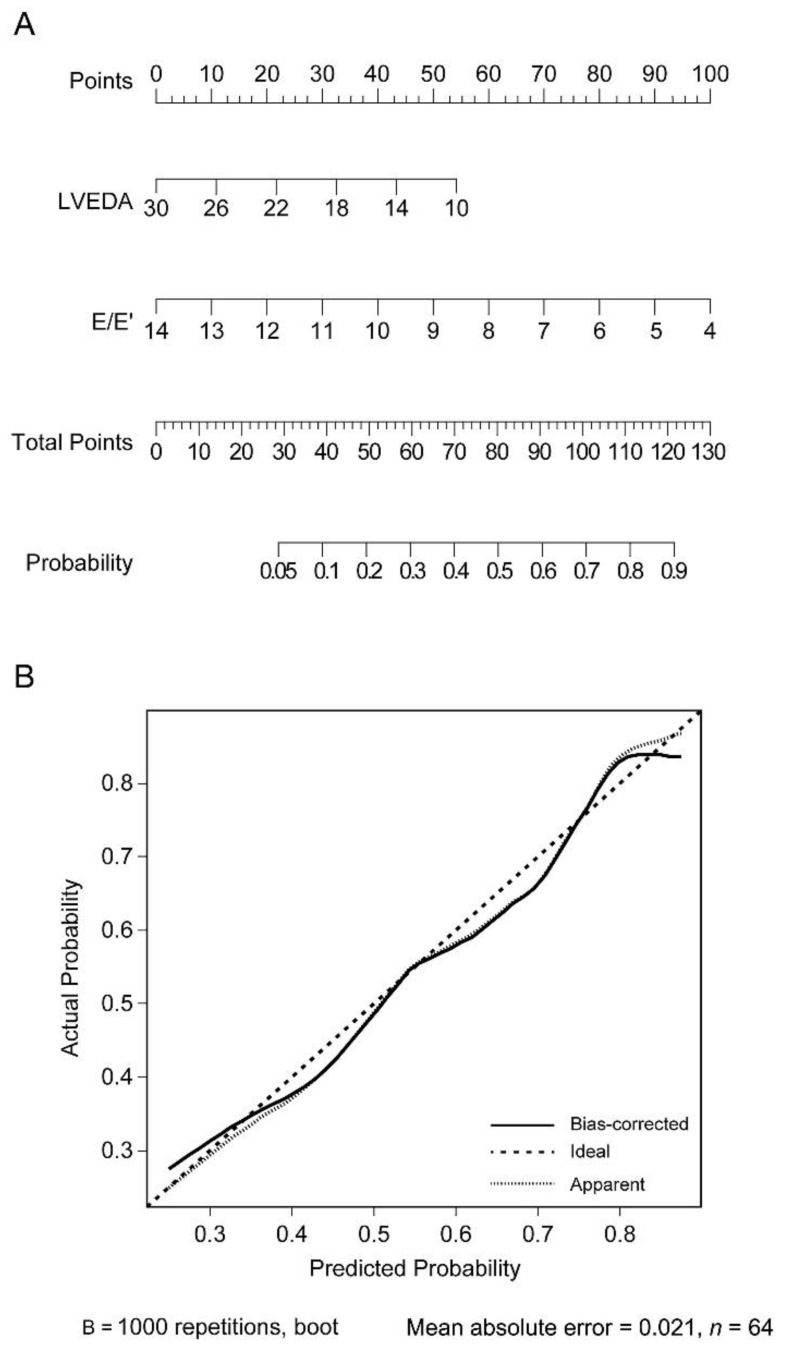
(**A**) Nomogram constructed with combination of left ventricular end-diastolic area (LVEDA) and the ratio of early mitral flow velocity to early diastolic velocity of the mitral annulus (E/E’). The *p* value of the Hosmer–Lemeshow goodness-of-fit test was 0.384. (**B**) Calibration plot comparing the actual and predicted probability of fluid responsiveness proposed by the nomogram with a mean absolute error of 0.021, *n* = 64.

Accordingly, a nomogram was constructed with LVEDA and E/E′ using a logistic regression model, which showed the highest AUROC (Figure 2A). The *p* value of the Hosmer–Lemeshow goodness-of-fit test was 0.384, indicating the adequacy of the constructed nomogram. The mean absolute error of the calibration plot of the nomogram was 0.021. This result indicates good correlation between the predicted probability proposed by the nomogram and the actual probability of fluid responsiveness (Figure 2B).

## 4. Discussion

In the current pilot study, we validated that the combination of static echocardiographic variables, LVEDA with E/E′, can predict fluid responsiveness; this combination showed the highest predictive ability among the concomitantly assessed preload indices, including PPV, in patients with preserved LV ejection fraction requiring surgical coronary revascularization.

In recent years, point-of-care ultrasound has become an important pillar of critical care as it can not only provide straightforward answers regarding some of the underlying causes of the hemodynamic instability but also guide fluid therapy [13]. In that context, echocardiographic dynamic indices, such as peak flow, velocity-time integral of the ascending aorta or carotid artery and diameter of the inferior or superior vena cava, have also emerged as useful predictors of fluid responsiveness [12,14]. However, these indices are also subject to the same inherent limitations related to heart–lung interaction [15]. By contrast, static preload indices, such as invasive filling pressures and non-invasive echocardiographic variables, can be readily and reliably assessed at end-expiration atmospheric pressure, regardless of the ventilatory status or heart rhythm of the patients. However, their predictive abilities for fluid responsiveness are absent as these indices lack information with regard to ventricular compliance and filling [7], which would exert significant influence on the ventricular response to a fluid challenge for the following reasons. The extent to which myocytes are stretched by a given preload is governed by muscle compliance [16]. Thus, the Frank–Starling mechanism and preload dependence would be significantly altered when myocyte extension is inadequate due to impaired relaxation or reduced distensibility [11].

For the investigation of diastolic function, TDI has become an invaluable tool and has been incorporated in the recommendations proposed by responsible societies in that regard [17,18]. TDI-derived E′ velocity tracks LV relaxation along the long axis and correlates well with the isovolumic LV relaxation rate [8]. As the major determinants of E velocity are left atrial pressure and LV relaxation rate, E/E′ corrected for LV relaxation was shown to estimate LV filling pressure in cardiac patients with preserved and reduced LV function [19,20]. Although less specific, E′/A′ has been proposed to reflect ventricular stiffness because stiffness is most pronounced in late diastole, in which A′ is reduced while E′ is less affected [9]. Moreover, E′/S′ has been proposed to reflect increased preload or systolic dysfunction considering the interdependence of these two variables and the compensatory increase in preload in case of low S′ [10,21]. In terms of assessing LV diastolic function, these Doppler-derived parameters fail to classify diastolic function in a fairly large number of patients as they may change acutely with altered loading conditions [22]. In contrast, these shortcomings of the Doppler-derived variables would theoretically be of great advantage in assessing fluid responsiveness as they would provide an instantaneous snapshot of LV diastolic mechanics at the time of assessment. Thus, we hypothesized that the combination of LVEDA and these parameters would act as a valuable preload index for predicting fluid responsiveness.

In the current study, only E/E′ and E′/S′ could discriminate fluid responders albeit with low predictive powers (AUROC of 0.71 and 0.68 for E/E′ and E′/S′, respectively), which are considered as poor for clinical use, while E’/A’ or LVEDA could not. Among the invasive preload indices, only PPV was able to predict fluid responsiveness, but at a lower predictive power (AUROC of 0.70) as well, whereas CVP and PAOP could not. Of the combinations, LVEDA and E/E′ yielded the highest predictive power (AUROC of 0.78), whereas the combinations of LVEDA and E′/S′ or E′/A′ could poorly predict fluid responsiveness (all AUROCs < 0.70), which may be attributable to the following. Global diastolic filling is mainly related to both early relaxation (active) and late elastic properties (passive) [23]. Although E and E′ all measure early diastolic events, E′ has also been validated to be related to elastic recoil, which governs passive relaxation [23,24], implicating that E′ may comprehensively reflect diastolic filling. As E velocity is highly dependent on loading conditions, mainly determined by the left atrial pressure and LV relaxation, E/E′ would yield sophisticated information related to the instantaneous LV compliance that may have imposed the incremental value for predicting fluid responsiveness when combined with LVEDA. A previous study outlined the ability of E/E′ alone, in the context of reflecting LV compliance, to predict fluid responsiveness in patients requiring surgical coronary revascularization, with an AUROC of 0.74, but its incremental value when combined with other parameters was not evaluated [25]. As indicated in the nomogram (Figure 2A), even an extremely low E/E′ value of 4 alone can only yield a total point of 100 corresponding to a 70% probability of being fluid responsive, while the addition of information obtained from LVEDA assessment can increase the probability to over 90% (the lower the values of LVEDA and E/E’, the more likely to be fluid responsive). By contrast, E′/A′ or E′/S′ is less specific and influenced by other factors, rendering their relationship with LV compliance less straightforward [9,10,21].

PPV is one of the most useful dynamic preload indices assuming that all of the conditions for proper heart–lung interaction are met [3]. However, in the current study, PPV’s predictive ability for fluid responsiveness was poor (AUROC of 0.70). Congruent to this result, previous studies have depicted that high preoperative E/E′ (>15) and pulse pressure hypertension (>60 mmHg), which are both closely related to diastolic dysfunction, resulted in abolished predictive ability of PPV or stroke volume variation in a similar subset of patients that require surgical coronary revascularization [6,26]. In the current study, 11 (17%) and 9 (14%) patients had high preoperative E/E′ and pulse pressure hypertension, respectively, which may have attributed to the low predictive ability of PPV.

In theory, this combined static index of LVEDA and E/E’ can potentially be applied to guide fluid therapy in clinical situations not suitable for the evaluation of dynamic indices or passive leg raising. However, being a pilot study, we measured these parameters in patients with sinus rhythm under closed chest condition, which merits further studies in a wide variety of patients in different clinical scenarios to prove its predictive ability on fluid responsiveness independent from heart-lung interaction or heart rhythm.

This study is subject to the following limitations. First, we used the lateral mitral annulus for TDI assessment. For simplicity, lateral E′ velocity is recommended given the potential influence of the right ventricle on septal E′ velocity [27]. However, the average of mitral annular velocities measured at different sites would have been the ideal method. Second, although the echocardiographic values were obtained during apnea at atmospheric pressure, anesthesia itself influences loading conditions. Thus, different cut-off values regarding E velocity and to a lesser extent E′ velocity are likely to be present in awake patients that need to be validated through further studies. Lastly, the difference in the incidence of diabetes and cerebrovascular accident between the responders and non-responders may have conveyed different influences on the vasculature and thus, diastology, which could not be expected and accounted for in this prospective, observational study.

## 5. Conclusions

In conclusions, the combined index of echocardiographic variables reflecting LV dimension (LVEDA) and diastolic compliance and filling (E/E′) can be a useful predictor of fluid responsiveness in anesthetized patients with preserved LV ejection fraction undergoing surgical coronary revascularization, showing better predictive ability than PPV in this subset of patients.

## Figures and Tables

**Table 1:** Patients’ characteristics.

	Responders (*n* = 40)	Non-Responders (*n* = 24)	*p*
Age (years)	66 ± 8	65 ± 8	0.577
Female	12 (30)	2 (8)	0.042
Body mass index (kg/m^2^)	25.3 ± 2.6	23.8 ± 3.1	0.037
Hypertension	24 (60)	17 (71)	0.382
Diabetes mellitus	12 (30)	15 (63)	0.011
Cerebrovascular accident	7 (18)	0 (0)	0.039
Myocardial infarction (<3 months)	4 (10)	2 (8)	1.000
Left main disease (>50% stenosis)	17 (43)	10 (42)	0.948
LVEF (%)	64 ± 6	67 ± 8	0.227
Medications			
Statin	27 (68)	19 (80)	0.315
Nitrate	16 (40)	10 (42)	0.895
Beta blocker	19 (48)	14 (58)	0.401
Calcium channel blocker	20 (50)	6 (25)	0.049
Renin-angiotensin system antagonist	23 (58)	12 (50)	0.560
Patients requiring norepinephrine	35 (88)	21 (88)	1.000
Plateau inspiratory pressure (cmH_2_O)	15 (14–16)	15 (14–16)	0.486

Note: Data are expressed as *n* (%), mean ± standard deviation or median (interquartile range). Abbreviation: LVEF, left ventricular ejection fraction.

**Table 2:** Hemodynamic variables and invasive preload indices at baseline and after fluid challenge.

	Responders (*n* = 40)			Non-Responders (*n* = 24)		
	Baseline	After Fluid Challenge	*p*	Baseline	After Fluid Challenge	*p*
HR (bpm)	59 ± 9	55 ± 6	<0.001	57 ± 5	55 ± 5	<0.001
MAP (mmHg)	72 ± 8	76 ± 9	<0.001	70 ± 8	76 ± 6	<0.001
MPAP (mmHg)	17 (15–19)	20 (18–22)	<0.001	16 (14–17)	19 (17–22)	<0.001
CI (L/min/m^2^)	2.1 (1.9–2.3)	2.6 (2.3–2.9) *	<0.001	2.3 (2.0–2.6)	2.1 (1.9–2.4)	<0.001
SVI (mL/beat/m^2^)	35 (32–38) *	46 (42–52) *	<0.001	38 (33–48)	37 (34–44)	<0.001
CVP (mmHg)	9 (8–11)	12 (10–13)	<0.001	9 (8–10)	11 (10–13)	<0.001
PAOP (mmHg)	13 (12–14)	16 (14–17)	<0.001	12 (10–14)	15 (13–18)	<0.001
PPV (%)	13 (8–14) *	4 (3–6) *	<0.001	9 (7–11)	6 (5–7)	<0.001

Note: Data are expressed as mean ± standard deviation or median (interquartile range). * Statistical significance between responders and non-responders. HR, heart rate; MAP, mean arterial pressure; MPAP, mean pulmonary arterial pressure; CI, cardiac index; SVI, stroke volume index; CVP, central venous pressure; PAOP, pulmonary artery occlusion pressure; PPV, pulse pressure variation.

**Table 3:** Echocardiographic preload indices at baseline and after fluid challenge.

		Baseline	After Fluid Challenge	*p*1 ^a^
LVEDA (cm^2^)	Responder	21.1 ± 3.8	23.9 ± 3.6	<0.001
	Non-responder	22.5 ± 3.0	23.9 ± 2.7	<0.001
	*p*2 ^b^	0.140	0.990	
E/E’	Responder	6.2 (5.6–7.0)	7.1 (6.1–8.0)	<0.001
	Non-responder	8.2 (6.1–9.2)	8.9 (7.4–9.8)	0.031
	*p*2	0.006	0.002	
E’/S’	Responder	0.9 ± 0.2	1.0 ± 0.2	0.004
	Non-responder	0.8 ± 0.2	0.9 ± 0.2	0.035
	*p*2	0.024	0.099	
E’/A’	Responder	1.3 (1.0–1.5)	1.4 (1.2–1.6)	0.013
	Non-responder	1.1 (0.9–1.4)	1.1 (0.9–1.5)	0.147
	*p*2	0.140	0.048	

Note: Data are expressed as mean ± standard deviation or median (interquartile range). ^a^ Statistical significance between baseline and after volume expansion. ^b^ Statistical significance between responders and non-responders. Abbreviations: LVEDA, left ventricular end-diastolic area; E, early transmitral flow velocity; E’, early-diastolic velocity of the lateral mitral annulus; S’, peak systolic velocity of the lateral mitral annulus; A’, late-diastolic velocity of the lateral mitral annulus.

**Table 4:** Receiver operating characteristic curves of indices of predicting fluid responsiveness.

	AUROC	95% CI	*p*
Invasive indices			
Central venous pressure	0.58	0.43–0.73	0.311
Pulmonary artery occlusion pressure	0.56	0.41–0.71	0.442
Pulse pressure variation	0.70	0.57–0.83	0.002
Echocardiographic indices			
Left ventricular end-diastolic area	0.60	0.46–0.74	0.170
E/E’	0.71	0.56–0.85	0.006
E’/S’	0.68	0.54–0.82	0.017
E’/A’	0.61	0.47–0.75	0.140
Combination of echocardiographic indices			
Left ventricular end-diastolic area with E/E’	0.78	0.66–0.90	<0.001
Left ventricular end-diastolic area with E’/S’	0.68	0.54–0.82	0.012
Left ventricular end-diastolic area with E’/A’	0.66	0.52–0.80	0.026

Abbreviations: AUROC, area under the receiver operating characteristic curve; CI, confidence interval; E, early transmitral flow velocity; E’, early-diastolic velocity of the lateral mitral annulus; S’, peak systolic velocity of the lateral mitral annulus; A’, late-diastolic velocity of the lateral mitral annulus.

## Data Availability

The data presented in this study are available on request from the corresponding author.

## References

[1] Marik P.E., Baram M., Vahid B. (2008). Does central venous pressure predict fluid responsiveness? A systematic review of the literature and the tale of seven mares. Chest.

[2] Boyd J.H., Forbes J., Nakada T.A., Walley K.R., Russell J.A. (2011). Fluid resuscitation in septic shock: A positive fluid balance and elevated central venous pressure are associated with increased mortality. Crit. Care Med..

[3] Marik P.E., Cavallazzi R., Vasu T., Hirani A. (2009). Dynamic changes in arterial waveform derived variables and fluid responsiveness in mechanically ventilated patients: A systematic review of the literature. Crit. Care Med..

[4] Michard F. (2005). Changes in arterial pressure during mechanical ventilation. Anesthesiology.

[5] Wyler von Ballmoos M., Takala J., Roeck M., Porta F., Tueller D., Ganter C.C., Schroder R., Bracht H., Baenziger B., Jakob S.M. (2010). Pulse-pressure variation and hemodynamic response in patients with elevated pulmonary artery pressure: A clinical study. Crit. Care.

[6] Kim S.Y., Song Y., Shim J.K., Kwak Y.L. (2013). Effect of pulse pressure on the predictability of stroke volume variation for fluid responsiveness in patients with coronary disease. J. Crit. Care.

[7] Osman D., Ridel C., Ray P., Monnet X., Anguel N., Richard C., Teboul J.L. (2007). Cardiac filling pressures are not appropriate to predict hemodynamic response to volume challenge. Crit. Care Med..

[8] Ommen S.R., Nishimura R.A., Appleton C.P., Miller F.A., Oh J.K., Redfield M.M., Tajik A.J. (2000). Clinical utility of doppler echocardiography and tissue doppler imaging in the estimation of left ventricular filling pressures: A comparative simultaneous doppler-catheterization study. Circulation.

[9] Zile M.R., Brutsaert D.L. (2002). New concepts in diastolic dysfunction and diastolic heart failure: Part I: Diagnosis, prognosis, and measurements of diastolic function. Circulation.

[10] Mogelvang R., Sogaard P., Pedersen S.A., Olsen N.T., Marott J.L., Schnohr P., Goetze J.P., Jensen J.S. (2009). Cardiac dysfunction assessed by echocardiographic tissue doppler imaging is an independent predictor of mortality in the general population. Circulation.

[11] Jacob R., Dierberger B., Kissling G. (1992). Functional significance of the frank-starling mechanism under physiological and pathophysiological conditions. Eur. Heart J..

[12] Song Y., Kwak Y.L., Song J.W., Kim Y.J., Shim J.K. (2014). Respirophasic carotid artery peak velocity variation as a predictor of fluid responsiveness in mechanically ventilated patients with coronary artery disease. Br. J. Anaesth..

[13] Denault A., Canty D., Azzam M., Amir A., Gebhard C.E. (2019). Whole body ultrasound in the operating room and intensive care unit. Korean J. Anesthesiol..

[14] Boyd J.H., Sirounis D., Maizel J., Slama M. (2016). Echocardiography as a guide for fluid management. Crit. Care.

[15] Sander M., Schneck E., Habicher M. (2020). Management of perioperative volume therapy-monitoring and pitfalls. Korean J. Anesthesiol..

[16] Levine H.J. (1972). Compliance of the left ventricle. Circulation.

[17] Nagueh S.F., Smiseth O.A., Appleton C.P., Byrd B.F., Dokainish H., Edvardsen T., Flachskampf F.A., Gillebert T.C., Klein A.L., Lancellotti P. (2016). Recommendations for the evaluation of left ventricular diastolic function by echocardiography: An update from the american society of echocardiography and the european association of cardiovascular imaging. J. Am. Soc. Echocardiogr..

[18] Mor-Avi V., Lang R.M., Badano L.P., Belohlavek M., Cardim N.M., Derumeaux G., Galderisi M., Marwick T., Nagueh S.F., Sengupta P.P. (2011). Current and evolving echocardiographic techniques for the quantitative evaluation of cardiac mechanics: ASE/EAE consensus statement on methodology and indications endorsed by the japanese society of echocardiography. J. Am. Soc. Echocardiogr..

[19] Rivas-Gotz C., Manolios M., Thohan V., Nagueh S.F. (2003). Impact of left ventricular ejection fraction on estimation of left ventricular filling pressures using tissue doppler and flow propagation velocity. Am. J. Cardiol..

[20] Kasner M., Westermann D., Steendijk P., Gaub R., Wilkenshoff U., Weitmann K., Hoffmann W., Poller W., Schultheiss H.P., Pauschinger M. (2007). Utility of doppler echocardiography and tissue doppler imaging in the estimation of diastolic function in heart failure with normal ejection fraction: A comparative doppler-conductance catheterization study. Circulation.

[21] Yip G.W., Zhang Y., Tan P.Y., Wang M., Ho P.Y., Brodin L.A., Sanderson J.E. (2002). Left ventricular long-axis changes in early diastole and systole: Impact of systolic function on diastole. Clin. Sci..

[22] Morris D.A., Belyavskiy E., Aravind-Kumar R., Kropf M., Frydas A., Braunauer K., Marquez E., Krisper M., Lindhorst R., Osmanoglou E. (2018). Potential usefulness and clinical relevance of adding left atrial strain to left atrial volume index in the detection of left ventricular diastolic dysfunction. JACC Cardiovasc. Imaging.

[23] De Boeck B.W., Cramer M.J., Oh J.K., van der Aa R.P., Jaarsma W. (2003). Spectral pulsed tissue doppler imaging in diastole: A tool to increase our insight in and assessment of diastolic relaxation of the left ventricle. Am. Heart J..

[24] Ohte N., Narita H., Hashimoto T., Akita S., Kurokawa K., Fujinami T. (1998). Evaluation of left ventricular early diastolic performance by color tissue doppler imaging of the mitral annulus. Am. J. Cardiol..

[25] Marques N.R., De Riese J., Yelverton B.C., McQuitty C., Jupiter D., Willmann K., Salter M., Kinsky M., Johnston W.E. (2019). Diastolic function and peripheral venous pressure as indices for fluid responsiveness in cardiac surgical patients. J. Cardiothorac. Vasc. Anesth..

[26] Shim J.K., Song J.W., Song Y., Kim J.H., Kang H.M., Kwak Y.L. (2014). Pulse pressure variation is not a valid predictor of fluid responsiveness in patients with elevated left ventricular filling pressure. J. Crit. Care.

[27] Mitter S.S., Shah S.J., Thomas J.D. (2017). A test in context: E/A and E/e’ to assess diastolic dysfunction and LV filling pressure. J. Am. Coll. Cardiol..

